# Pseudonocardians A–C, New Diazaanthraquinone Derivatives from a Deap-Sea Actinomycete *Pseudonocardia* sp. SCSIO 01299

**DOI:** 10.3390/md9081428

**Published:** 2011-08-23

**Authors:** Sumei Li, Xinpeng Tian, Siwen Niu, Wenjun Zhang, Yuchan Chen, Haibo Zhang, Xianwen Yang, Weimin Zhang, Wenjun Li, Si Zhang, Jianhua Ju, Changsheng Zhang

**Affiliations:** 1 CAS Key Laboratory of Marine Bio-resources Sustainable Utilization, RNAM Center for Marine Microbiology, Guangdong Key Laboratory of Marine Materia Medica, South China Sea Institute of Oceanology, Chinese Academy of Sciences, Guangzhou 510301, China; E-Mails: lism@scsio.ac.cn (S.L.); xinpengtian@yahoo.com.cn (X.T.); niusi123@126.com (S.N.); wenjunzha@gmail.com (W.Z.); zhanghb@scsio.ac.cn (H.Z.); yangxw76@163.com (X.Y.); zhsimd@scsio.ac.cn (S.Z.); jju@scsio.ac.cn (J.J.); 2 Guangdong Institute of Microbiology, 100 Central Xianlie Road, Guangzhou 510070, China; E-Mails: yuchan2006@126.com (Y.C.); wmzhang58@yahoo.com.cn (W.Z.); 3 Yunnan Institute of Microbiology, Yunnan University, Kunming 650091, China; E-Mail: wjli@ynu.edu.cn

**Keywords:** marine actinomycetes, *Pseudonocardia*, natural products, cytotoxicity, antibacterial, South China Sea

## Abstract

Pseudonocardians A–C (**2**–**4**), three new diazaanthraquinone derivatives, along with a previously synthesized compound deoxynyboquinone (**1**), were produced by the strain SCSIO 01299, a marine actinomycete member of the genus *Pseudonocardia*, isolated from deep-sea sediment of the South China Sea. The structures of compounds **1**–**4** were determined by mass spectrometry and NMR experiments (^1^H, ^13^C, HSQC, and HMBC). The structure of compound **1**, which was obtained for the first time from a natural source, was confirmed by X-ray analysis. Compounds **1**–**3** exhibited potent cytotoxic activities against three tumor cell lines of SF-268, MCF-7 and NCI-H460 with IC_50_ values between 0.01 and 0.21 μm, and also showed antibacterial activities on *Staphylococcus aureus* ATCC 29213, *Enterococcus faecalis* ATCC 29212 and *Bacillus thuringensis* SCSIO BT01, with MIC values of 1–4 μg mL^−1^.

## 1. Introduction

Marine microorganisms, especially marine actinomycetes, are continuing to be rich sources of bioactive metabolites, with 273 new compounds reported from marine microbes in 2009 [1]. Natural products derived from marine actinomycetes displayed a wide range of bioactivities, such as antitumor [2,3], antiinfective [4], and antimalarial [5]. In recent years, South China Sea has been emerging as a potentially abundant source of novel species/genera of marine actinomycetes [6–10]. Some new bioactive compounds, such as marinactinones A–C [10], lobophorins E and F [11], were reported from marine actinomycetes isolated from the South China Sea.

In our continuous search for bioactive secondary metabolites from South China Sea-derived actinomycetes, crude extracts of the strain SCSIO 01299 were found to exhibit significant cytotoxic and antibacterial activities. Upon large fermentation of the strain SCSIO 01299, four products (**1**–**4**) were isolated and the main product **1** showed UV absorptions at 276, 354, 461 nm, characteristic of a highly conjugated chromophore related to anthraquinone compounds [12]. Herein we report the preliminary characterization of the strain SCSIO 01299 as a member of the genus *Pseudonocardia* by 16S rRNA gene sequence analysis, the isolation and structural elucidation of four diazaanthraquinone derivatives including a previously synthesized compound deoxynyboquinone (DNQ, **1**) and three new analogues, pseudonocardians A–C (**2**–**4**). In addition, antibacterial and cytotoxic activities for compounds **1**–**4** were investigated.

## 2. Results and Discussion

### 2.1. Taxonomy of the Producing Strain

**Figure 1.**
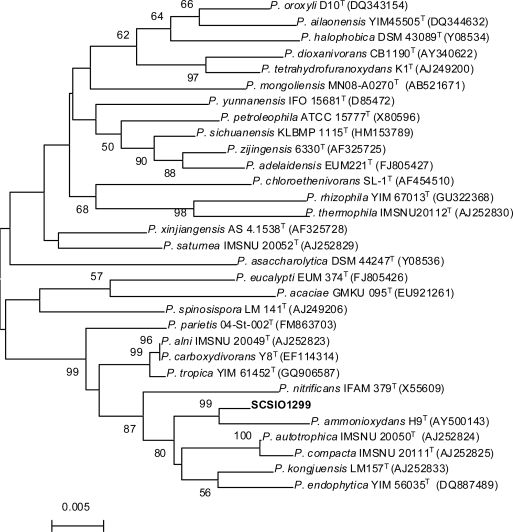
Phylogenetic dendrogram of the strain SCSIO 01299 and its closest relatives reconstructed by the neighbor-joining method based on 16S rRNA gene sequences.

The strain SCSIO 01299 was isolated from deep-sea sediment (−3258 m) of the South China Sea, and was preliminarily identified as an actinomycetal species based on morphology observation. The 16S rRNA gene of the strain SCSIO 01299 was PCR amplified, sequenced and submitted to GenBank (accession number is JN204514). BLAST results showed that the new isolate had the highest similarity (98%) with *Pseudonocardia autotrophica* IMSNU 20050^T^ [13]. The phylogenetic tree generated by a neighbor-joining method clearly revealed the evolutionary relationship of the strain SCSIO 01299 to a group of *Pseudonocardia* species (Figure 1). Thus, this strain was designated *Pseudonocardia* sp. SCSIO 01299.

### 2.2. Structural Elucidation

**Figure 2.**
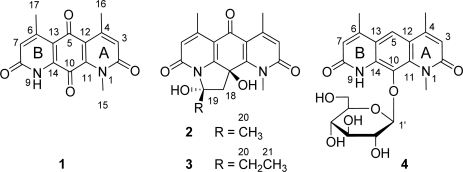
Chemical structures of compounds **1**–**4**.

**Figure 3.**
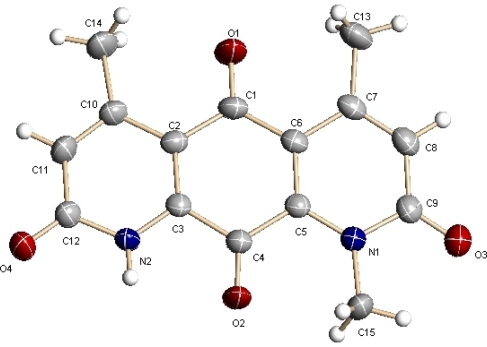
X-ray analysis of compound **1**.

Compound **1** was obtained as red needles. It gave a [M + H]^+^ at *m/z* 285.0 and a [M − H]^−^ at *m/z* 283.2 in the ESI-MS, indicating a molecular weight of 284.0. The ^1^H and ^13^C NMR spectra of **1** displayed 15 carbon signals, including two methyl doublets [δ_H_ 2.55 (3H, d, *J* = 1.0 Hz, Me-17), 2.59 (3H, d, *J* = 1.0 Hz, Me-16); δ_C_ 22.1 (q, Me-17), 23.0 (q, Me-16)] and one methyl singlet [δ_H_ 4.01 (3H, s, Me-15); δ_C_ 33.9 (q, Me-15)], two sp^2^ methines [δ_H_ 6.78 (1H, d, *J* = 1.0 Hz, H-7), 6.82 (1H, d, *J* = 1.0 Hz, H-3); δ_C_ 126.8 (d, C-3) and 127.1 (d, C-7)], and 10 sp^2^ quarternary carbons with four from carbonyls [δ_C_ 161.4 (s), 162.4 (s), 182.4 (s), and 176.9 (s)] (Table 1). The structure of ring A in **1** was constructed based on HMBC correlations of the methyl H_3_-15 to C-2/C-11, the methyl H_3_-16 to C-3/C-4/C-12, and H-3 to C-2/C-12/C-16. The structure of ring B was deduced from HMBC correlations of H-7 to C-8/C-13/C-17 and H_3_-17 to C-6/C-7/C-13. These two moieties were then connected through two carbonyls [δ_C_ 182.4 and 176.9]. Finally, **1** was unambiguously identified to be deoxynyboquinone (DNQ, Figures 2 and 3), a chemically synthesized compound [14,15], by X-ray crystallographic analysis.

**Figure 4.**
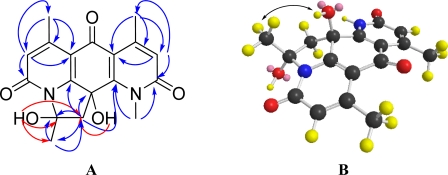
Two dimensional NMR characterizations of compound **2**: (**A**) Selected HMBC correlations; (**B**) Key NOESY correlation and mimic structure of **2**, using the MM2 minimum energy calculation by ChemBio3D Ultra 11.0.

Compound **2**, designated pseudonocardian A, was isolated as a white solid. The molecular formula of compound **2** was established as C_18_H_18_N_2_O_5_ (*m/z* 343.1305, calculated for 343.1294 [M + H]^+^), indicating 11 degrees of unsaturation. The ^1^H and ^13^C NMR spectra of compound **2** were similar to those of **1**, except that the C-10 carbonyl in **1** was absent in **2**. Instead, more modifications on C-10 were found in **2**, including two oxygenated quaternary carbons [δ_C_ 72.7 (s, C-10), 97.6 (s, C-19)], a methylene [δ_H_ 2.71 (d, *J* = 13.5 Hz, H-18), 3.30 (d, *J* = 13.5 Hz, H-18), δ_C_ 52.4 (t, C-18)] and a methyl singlet [δ_H_ 2.19 (s, Me-20), δ_C_ 26.5 (q, Me-20)]. Taking the unsaturation degrees into consideration, there should be an additional ring in **2**. Based on the HMBC correlations of H-18 to C-10/C-11/C-14/C-19, and of H-20 to C-18/C-19 (Figure 4A), C-19 was supposed to be connected to N-9. The assumption was confirmed by the downfield shift of C-19 at δ_C_ 97.6. After careful analysis of HMBC correlations, the planar structure of **2** was established (Figure 2). In order to assign the relative configuration of **2**, a NOESY experiment was carried out in DMSO-*d*_6_. NOESY correlation of OH-10 to H_3_-20 was found (Figure 4B), indicating the two hydroxyls (OH-10 and OH-19) were on the opposite sides. This was consistent with the mimic configuration using the MM2 minimum energy calculation by ChemBio3D Ultra 11.0 (Figure 4B). Therefore, the relative configuration of compound **2** was shown in Figure 2.

Compound **3**, designated pseudonocardian B, was obtained as white solid. Its molecular formula was assigned as C_19_H_20_N_2_O_5_ based on the HR-MS data (*m/z* 357.1456, calculated for 357.1450, [M + H]^+^). Comparing the ^1^H and ^13^C NMR spectroscopic data of **3** with **2**, the only difference was that the C-19 methyl singlet in **2** was substituted by an ethyl group [δ_H_ 1.22 (t, *J* = 7.5 Hz, Me-21), 2.41 (dd, *J* = 7.5, 14.0 Hz, H-20a), 2.84 (dd, *J* = 7.5, 14.0 Hz, H-20b), δ_C_ 9.3 (q, Me-21), 31.6 (t, C-20)] in **3** (Table 1). This substitution was confirmed by HMBC correlations of H-21 to C-19/C-20 and of H-20 to C-18/C-19/C-21. Therefore, the structure of **3** was established as shown in Figure 2.

Compound **4**, designated pseudonocardian C, was isolated as a red brown powder. The molecular formula of **4** was established as C_21_H_24_N_2_O_8_ by HR-MS (*m/z* 433.1609, calculated for 433.1611 [M + H]^+^). In comparison of the ^1^H and ^13^C NMR spectroscopic data of **4** and **1**, signals for both ring A [δ_H_ 6.57 (s, H-3), δ_C_ 166.0 (s, C-2), 151.4 (s, C-2), 137.3 (s, C-11), 120.8 (d, C-3), 118.7 (s, C-12)] and ring B [δ_H_ 6.54 (s, H-7), δ_C_ 164.9 (s, C-8), 149.4 (s, C-6), 135.1 (s, C-14), 120.4 (d, C-7), 120.4 (s, C-13)] were found in **4**. However, the two ketone groups (C-5 and C-10) in **1** were displaced in **4** by a sp^2^ methine singlet [δ_H_ 8.01 (s, H-5), δ_C_ 119.7 (d, C-5)], an oxygenated sp^2^ quaternary carbon signals [δ_C_ 132.1 (s, C-10)], and a β-glucose moiety with coupling constant *J*_1′,2′_ of 8.0 Hz [16]: [δ_H_ 4.57 (1H, d, *J* = 8.0 Hz, H-1′), 3.66 (1H, m, H-2′), 3.44 (1H, m, H-3′), 3.46 (1H, m, H-4′), 3.13 (m, H-5′), 3.73 (1H, dd, *J* = 2.3, 11.8 Hz, H-6a′), 3.68 (1H, dd, *J* = 5.0, 11.8 Hz, H-6b′); δ_C_ 107.8 (d, C-1′), 75.5 (d, C-2′), 78.0 (d, C-3′), 70.9 (d, C-4′), 78.7 (d, C-5′), 62.3 (t, C-6′)]. The HMBC correlation from H-5 to C-4/C-6/C-11/C-14 indicated that moiety A and moiety B were connected by a benzene ring. And the HMBC correlation from H-1 to C-10 showed that the glucose was located at C-10. On these bases of these cumulative evidences, the structure of **4** was established as shown in Figure 2.

### 2.3. Biological Activities

Compounds **1**–**4** were evaluated for their antibacterial activities against *Staphylococcus aureus* ATCC 29213, *Enterococcus faecalis* ATCC 29212 and *Bacillus thuringensis* SCSIO BT01, and *in vitro* cytotoxic activities against three human tumor cell lines, including SF-268 (human glioma cell line), MCF-7 (human breast adenocarcinoma cell line) and NCI-H460 (human non-small cell lung cancer cell line) (Table 2). Compounds **1**–**3** showed strong antibacterial activities towards all three assayed indicators with MIC values ranging from 1–4 μg mL^−1^. Pseudonocardian A (**2**), bearing a methyl group at C-19, displayed one-fold less antibacterial activities than pseudonocardian B (**3**), which contained an ethyl group at C-19. DNQ (**1**), pseudonocardians A (**2**) and B (**3**) exhibited nearly the same potency against tumor cell lines SF-268 and MCF-7 with IC_50_ values in the range of 15–28 nM (Table 2). DNQ (**1**) showed a slightly better activity than pseudonocardians A (**2**) and B (**3**) against NCI-H460 (Table 2). Pseudonocardian C (**4**) showed no antibacterial activity, while preserved certain *in vitro* cytotoxic activities comparable to those of the control compound cisplatin. However, its cytotoxicities were largely reduced when compared to **1**–**3** (Table 2).

### 2.4. Discussion

Diazaanthraquinones comprise a class of natural products, either naturally-occurring [17–22], or chemically synthesized [23–30], both of them exhibited good antitumor or antibacterial activities, highlighting their potential for drug development. Deoxynyboquinone (DNQ, **1**), a diazaanthraquinone, was originally synthesized during nybomycin structural studies [14]. Recently, a facile synthetic route for DNQ (**1**) was reported [15]. Studies on biological activity and mechanism of action revealed that DNQ (**1**) rivaled doxorubicin for the potency in tumor cell culture and was even effective against a doxorubincin-resistant cell line, with a mechanism of inducing cell death through generation of reactive oxygen species (ROS) [15].

In this study, we reported the isolation of DNQ (**1**) for the first time from a natural source, the marine actinomycete *Pseudonocardia* sp. SCSIO 01299. In addition, we confirmed the structure of DNQ (**1**) by X-ray crystallographic analysis for the first time. More interestingly, we isolated 3 novel DNQ (**1**) analogues, pseudonocardians A–C (**2**–**4**), from the same strain. Consistent with previous report [15], DNQ **(1**) exhibited potent *in vitro* cytotoxic activities against three tumor cell lines of SF-268, MCF-7 and NCI-H460, with IC_50_ values of 22, 15, and 80 nM, respectively. Pesudonocardians A (**2**) and B (**3**) had almost the same *in vitro* cytotoxic activities as those of DNQ (**1**), with the added benefit of being ∼10-fold more aqueously soluble than **1**. Pseudonocardian C (**4**), although being >300-fold less potent than **1**–**3**, showed comparable cytotoxicity to the positive control cisplatin (Table 2). Unlike **1**–**3**, pseudonocardian C (**4**) displayed no observable antibacterial activities, probably due to the glucosylation at C-10, which resembled the inactivation of macrolide antibiotics by glycosylation as a resistance mechanism [31].

## 3. Experimental Section

### 3.1. General Experimental Procedures

Materials for column chromatography (CC) were silica gel (100–200 mesh; 300–400 mesh; Jiangyou Silica Gel Development, Inc., Yantai, China), Sephadex LH-20 (40–70 μm; Amersham Pharmacia Biotech AB, Uppsala, Sweden), and YMC-GEL ODS-A (12 nm S-50 μm; Japan). Thin layer chromatography (TLC, 0.1–0.2 mm or 0.3–0.4 mm) was conducted with precoated glass plates (silica gel GF_254_, 10–40 nm, Jiangyou Silica Gel Development, Inc., Yantai, China). Medium pressure liquid chromatography (MPLC) was performed on automatic flash chromatography (EZ Purifier III, Leisure Science Co., Ltd. Shanghai, China) with monitor wavelength of 234 nm and collector wavelength of 300 nm. Mass spectral data were obtained on a quadrupole-time-of-flight mass spectrometry (Waters, Milford, MA, USA) for high resolution fast atom bombardment mass spectrometry (HRFABMS). The optical rotation was recorded on a 341 polarimeter (Perkin Elmer, Inc., Norwalk, CT, USA). ^1^H, ^13^C NMR and 2D NMR spectra were recorded on a Bruker AV-500 MHz NMR spectrometer (Bruker Biospin GmbH, Rheinstetten, Germany) with tetramethylsilane (TMS, δ 0.0 ppm) as the internal standard. ^1^H NMR data were reported as chemical shift (multiplicity [singlet (s), doublet (d), triplet (t), and multiplet (m)], and coupling constants (Hz); ^13^C NMR data were reported as follows: chemical shift [quaternary carbon (s), methine (d), methylene (t), methyl (q)]. Deuterated NMR solvents were purchased from Cambridge Isotopes (Andover, MA, USA).

### 3.2. Microbiological Material

The strain SCSIO 01299 was isolated from a sediment sample (E 120°0.975′, N 19°0.664′) at the depth of 3258 m collected from an open voyage to the South China Sea in August 2007, and was deposited in the type culture collection of Center for Marine Microbiology, Research Network of Applied Microbiology, South China Sea Institute of Oceanology, Chinese Academy of Sciences, Guangzhou, China. Genomic DNA isolation, PCR amplification of 16S rRNA gene, sequence alignment, and phylogenetic tree construction of the strain SCSIO 01299 were performed as described previously [6]. A single colony of SCSIO 01299 was inoculated into 50 mL seed medium (soybean meal 0.5%, soluble starch 1.5%, peptone bacteriological 1.5%, glycerol 1.5%, CaCO_3_ 0.2%, sea salt 3%, pH 7.4, adjusted before sterilization) in 250 mL Erlenmeyer flasks, and was cultured on a rotary shaker at 200 r.p.m. and 28 °C for 2 days. 10% inoculums were transferred into 50 mL production medium (the same as the seed medium) in 250 mL Erlenmeyer flasks, and were subsequently incubated on a rotary shaker at 200 r.p.m., 28 °C for 5 days.

### 3.3. Extraction and Isolation

The fermentation broth (9 L) was extracted with equal volume of butanone for 4 times to afford the residue A after evaporation. The mycelia cake was extracted 3 times with 6 L acetone. After removing acetone, the residue was re-extracted by 6 L butanone to afford the residue B upon removal of the solvent under vacuum. Residues A and B were combined and subjected to column chromatography (CC) over silica gel (300–400 mesh, 150 g), eluting with a gradient of CHCl_3_/CH_3_OH (100:0→0:100) to give three fractions (Fr.1–Fr.3). Compound **1** (150.3 mg, 16.7 mg L^−1^) was obtained from fraction Fr.1 after elution with CHCl_3_/CH_3_OH (1:1) on Sephadex LH-20 and repeated recrystallization in CHCl_3_/CH_3_OH (10:1). Fraction Fr.2 was subjected to Sephadex LH-20 column chromatography, eluting with CHCl_3_/CH_3_OH (1:1), and then further chromatographed on silica gel (300–400 mesh, 40 g), eluting with CHCl_3_/CH_3_OH (20:1). Final purification was conducted by C18 reverse phase MPLC (20 × 2.5 cm ID), eluting with a linear gradient of CH_3_OH/H_2_O (0%→55%, 15 mL min^−1^, 100 min), to afford **2** (15.8 mg, 1.76 mg L^−1^) and **3** (12.5 mg, 1.39 mg L^−1^). Similarly, **4** (6.6 mg, 0.73 mg L^−1^) was obtained from Fr.4 after column chromatography on Sephadex LH-20 (CHCl_3_/CH_3_OH, 1:1) and further purification by RP-MPLC.

### 3.4. Characterization Data

Deoxynyboquinone (**1**). Red needles, UV (CH_3_CN/H2O/TFA) λ_max_: 276, 354, 461 nm. ^1^H and ^13^C NMR data, see Table 1; ESIMS *m/z* 285.0 [M + H]^+^, 569.7 [2M + H]^+^, 283.2 [M − H]^−^, 567.4 [2M − H]^−^.

Crystal data for deoxynyboquinone (**1**) [32]: Red triclinic crystal of C_15_H_12_N_2_O_4_. Space group P-1, *a* = 4.8353 (10) Å, *α* = 85.195(4)°; *b* = 9.373(2) Å, *β* = 89.350(5)°; *c* = 13.780 (3) Å, *γ* = 82.094(4)°; *V* = 616.5 (2) Å^3^, Z = 2; crystal size 0.301 × 0.126 × 0.057 mm^3^. A total of 3289 unique reflections (*θ* = 2.20–25.49°) were collected using graphite monochromated Mo K*α* (*λ* = 0.71073 Å) on a CCD area detector diffractometer. The structure was solved by direct methods (SHELXS-97) and expanded using Fourier techniques (SHELXS-97). The final cycle of full-matrix least-squares refinement was based on 2273 data, 0 restraints and 198 variable parameters. Final R indicates R_1_ = 0.0690, wR_2_ = 0.1725 [I > 2σ(I)].

Pseudonocardian A (**2**). White solid, ${\left[\alpha \right]}_{D}^{20}$ +1.52° (*c* 0.46, MeOH), UV (CH_3_CN/H2O/TFA) λ_max_: 235, 300 nm. ^1^H and ^13^C NMR data, see Table 1. HRESI-MS [M + H]^+^ *m/z* 343.1305 (calcd. for C_18_H_19_N_2_O_5_ 343.1294), ESIMS: *m/z* 343.2 [M + H]^+^, 685.4 [2M + Na]^+^, 341.1 [M − H]^−^, 683.3 [2M − H]^−^.

Pseudonocardian B (**3**). White solid, ${\left[\alpha \right]}_{D}^{20}$ −1.56° (*c* 0.90, MeOH), UV (CH_3_CN/H2O/TFA) λ_max_: 235, 300 nm. ^1^H and ^13^C NMR data, see Table 1. HRESI-MS: *m/z* [M + H]^+^ 357.1456 (calcd. for C_19_H_21_N_2_O_5_, 357.1450); ESIMS: *m/z* 357.2 [M + H]^+^, 379.3 [M + Na]^+^, 735.3 [2M + Na]^+^, 355.2 [M − H]^−^, 711.0 [2M − H]^−^.

Pseudonocardian C (**4**). Red brown powder, ${\left[\alpha \right]}_{D}^{25}$ −25.6° (*c* 0.16, MeOH), UV (CH_3_CN/H2O/TFA) λ_max_: 236, 264, 352, 368 nm. ^1^H NMR (500 MHz, CD_3_OD): 8.01 (1H, s, H-5), 6.57 (1H, s, H-3), 6.54 (1H, s, H-7), 4.57 (1H, d, *J* = 8.0 Hz, H-1′), 3.96 (1H, s, H-15), 3.73 (1H, dd, *J* = 2.3, 11.8 Hz, H-6′a), 3.68 (1H, dd, *J* = 5.0, 11.8 Hz, H-6′b), 3.66 (1H, m, H-2′), 3.46 (1H, m, H-4′), 3.44 (1H, m, H-3′), 3.13 (1H, m, H-5′), 2.63 (1H, s, H-16), 2.60 (1H, s, H-17); and ^13^C NMR (125 MHz, CD_3_OD): 166.0 (s, C-2), 164.9 (s, C-8), 151.4 (s, C-6), 149.4 (s, C-4), 137.3 (s, C-11), 135.1 (s, C-14), 132.1 (s, C-10), 120.8 (d, C-3), 120.4 (d, C-7), 120.4 (s, C-13), 119.7 (d, C-5), 118.7 (s, C-12), 107.8 (d, C-1′), 78.7 (d, C-5′), 78.0 (d, C-3′), 75.5 (d, C-2′), 70.9 (d, C-4′), 62.3 (t, C-6′), 37.4 (q, Me-15), 19.3 (q, Me-17), 19.0 (q, Me-16). HRESI-MS *m/z* 433.1609 [M + H]^+^ (calcd. for C_21_H_25_N_2_O_8_, [M + H]^+^ 433.1611); ESIMS *m/z* 433.2 [M + H]^+^, 455.4 [M + Na]^+^, 887.2 [2M + Na]^+^, 431.3 [M − H]^−^, 467.2 [M + Cl]^−^, 863.3 [2M − H]^−^.

### 3.5. Antibacterial and Cytotoxic Assay

Minimal Inhibition Concentration (MIC) values of deoxynyboquinone (**1**) and pseudonocardians A–C (**2**–**4**) were determined against 3 indicators (*Staphylococcus aureus* ATCC 29213, *Enterococcus faecalis* ATCC29212 and *Bacillus thuringiensis* SCSIO BT01), according to previously described methods [33]. Cytotoxicities of the four compounds were assayed against 3 tumor cell lines, including MCF-7 (human breast adenocarcinoma cell line), NCI-H460 (human non-small cell lung cancer cell line), and SF-268 (human glioma cell line). Assays were performed as described previously [34].

## 4. Conclusions

We have found that deep-sea actinomycete *Pseudonocardia* sp. SCSIO 01299 is a natural producer of the promising anticancer drug candidate deoxynyboquinone (**1**) and three diazaanthraquinone derivatives pseudonocardians A–C (**2**–**4**). In comparison with **1**, pseudonocardians A (**2**) and B (**3**) showed the same anticancer potency but had enhanced aqueous solubility. These findings once again highlighted the potential of marine actinomycetes for novel drug discovery.

## Figures and Tables

**Table 1.** ^1^H and ^13^C NMR spectroscopic data of compounds **1**–**3**.

**No.**	**1***^a^*		**2***^b^*		**2***^c^*		**3***^b^*	

	**δ_H_(*J*in Hz)**	**δ_C_**	**δ_H_(*J*in Hz)**	**δ_C_**	**δ_H_(*J*in Hz)**	**δ_C_**	**δ_H_(*J*in Hz)**	**δ_C_**
2		161.4 s		164.5 s		161.0 s		164.5 s
3	6.82 d (1.0)	126.8 d	6.48, s	121.4 d	6.39, d (1.0)	119.8 d	6.49, d (1.0)	121.4 d
4		149.0 s		153.5s		150.6 s		153.5 s
5		182.4 s *		183.5 s		181.9 s		183.6 s
6		150.5 s		153.8 s		150.1 s		153.6 s
7	6.78 d (1.0)	127.1 d	6.32, s	122.6 d	6.24, d (1.0)	121.2 d	6.30, d (1.0)	122.6 d
8		162.4 s		162.8 s		159.3 s		163.0 s
10		176.9 s *		72.7 s		70.8 s		72.8 s
11		141.4 s		151.8 s		149.9 s		152.0 s
12		118.2 s		117.8 s		114.7 s		117.8 s
13		114.9 s		110.8 s		107.8 s		111.0 s
14		141.7 s		157.0 s		155.8 s		156.9 s
15	4.01, s	33.9 q	3.90, s	34.1 q	3.71, s	32.6 q	3.90, s	34.2 q
16	2.59, d (1.0)	23.0 q	2.57, s	23.6 q	2.44, d (1.0)	22.7 q	2.55, d, (1.0)	23.5 q
17	2.55, d (1.0)	22.1 q	2.58, s	21.3 q	2.45, d (1.0)	20.5 q	2.59, d (1.0)	21.3 q
18			2.71, d (13.5),	52.4 t	2.62, d (13.5),	50.8 t	2.51, d (13.8),	47.9 t
			3.30, d (13.5)		3.13, d (13.5)		3.41, d (13.8)	
19				97.6 s		95.5 s		100.8 s
20			2.19, s	26.5 q	2.03, s	25.9 q	2.41, dd (7.5, 14.0),	31.6 t
							2.84, dd (7.5, 14.0)	
21							1.22, t (7.5)	9.3 q

* Exchangeable.

a Measured in pyridine-*d*_5_;

b Measured in CD_3_OD;

c Measured in DMSO-*d*_6_. 500 MHz for ^1^H NMR; 125 MHz for ^13^C NMR.

**Table 2.** Antibacterial and cytotoxic activities of compounds **1**–**4**.

**Compounds**	**MIC (μg mL^–1^)**			**IC_50_(μM)**		
	***B. thuringensis*SCSIO BT01**	***S. aureus*ATCC 29213**	***E. faecalis*ATCC 29212**	**SF-268**	**MCF-7**	**NCI-H460**
**1**	1	1	1	0.022	0.015	0.080
**2**	4	4	2	0.028	0.027	0.209
**3**	2	2	2	0.022	0.021	0.177
**4**	>128	>128	>128	6.70	8.02	43.28
**Cisplatin**	ND *^a^*	ND *^a^*	ND *^a^*	3.99	9.24	1.53

a Not detected.
